# Beyond the hype: a comprehensive exploration of CBD’s biological impacts and mechanisms of action

**DOI:** 10.1186/s42238-025-00274-y

**Published:** 2025-05-11

**Authors:** Karli Swenson

**Affiliations:** Department of Pediatrics, University of Colorado Anschutz Medical Campus, 13123 East 16 Ave B265, Aurora, CO 80045 USA

**Keywords:** Cannabidiol, CBD, Cannabinoids, Mechanism of Action, Receptor Activation

## Abstract

**Background:**

Cannabidiol (CBD) is the primary non-psychoactive component of cannabis. Consumption of CBD is increasing rapidly as it is federally legal and widely available in the United States, Europe, Mexico, Canada, and Asia. CBD is gaining traction in medical and biochemical research, though a comprehensive classification of CBD receptor interactions is yet to be elucidated.

**Methods:**

A comprehensive literature search across PubMed, Web of Science, and Google Scholar identified studies reporting cannabidiol (CBD) interactions with receptors, enzymes, and biological processes. Eligible articles included cell culture, animal model, biochemical, and clinical studies. Findings were thematically synthesized by body system, emphasizing mechanisms and implications for health and disease.

**Results:**

Herein, I compile the literature to date of known interactions between CBD and various receptors, enzymes, and processes. I discuss the impact of CBD exposure on multiple processes, including endocannabinoid receptors, ion channels, cytochrome 450 enzymes, inflammatory pathways, and sex hormone regulation. I explain the potential effects of CBD on psychiatric disorders, seizure activity, nausea and vomiting, pain sensation, thermal regulation, neuronal signaling, neurodegenerative diseases, reproductive aging, drug metabolism, inflammation, sex hormone regulation, and energy homeostasis.

**Conclusions:**

Understanding how CBD functions and how it can interact with other recreational or pharmaceutical medications is necessary for proper clinical management of patients who consume CBD.

## Introduction

Cannabis consumption is increasing rapidly in tandem with increased legalization and availability and decreased social stigma (Patrick et al. 2022). As of 2023, recreational cannabis consumption is legal in 24 states and medicinal cannabis is available in an additional 17 states in the United States (Marijuana legality by state - Updated Oct 1, 2023. DISA 2025). In the United States, roughly 18% of the adult population report consuming cannabis, making it the most consumed federally illicit substance (Results from the 2019 National Survey on Drug Use and Health (NSDUH): Key Substance Use and Mental Health Indicators in the United States | SAMHSA Publications and Digital Products n.d.). Cannabis products contain multiple component parts, including tetrahydrocannabinol (THC), cannabidiol (CBD), and minor cannabinoids and terpenes (Atakan 2012). CBD was removed from the federal schedule 1 drug classification in 2018 (Abernethy 2019) and is now widely available in gas stations and grocery stores in all U.S. states. CBD consumption is dramatically increasing both recreationally and medicinally (Goodman et al. 2022). CBD has multiple medicinal effects, including as a nausea reducing medication, an anxiety reducing medication, a sleep aid (Rapin et al. 2021), and one pharmaceutical CBD product, Epidiolex, is approved by the Food and Drug Administration (FDA) to treat severe childhood seizure (Abu-Sawwa et al. 2020). One inherent limitation with CBD research is the challenge of sourcing product that is pure, reliable, transparent in dosing, and available for various administration routes. As the landscape of synthetic and hemp-derived CBD product changes in the early 2020’s, access to product for research is an actively evolving field. Cannabidiol (CBD) has emerged as a highly popular and rapidly evolving area of research, with several comprehensive reviews published in recent years that explore its pharmacological properties and therapeutic potential. Notable reviews, such as those by Sideris and Doan (2024), Castillo-Arellano et al. (2023), and Vitale et al. (2021), have provided valuable insights into CBD’s effects, particularly its polypharmacological actions in neuropsychiatric conditions (Sideris and Doan 2024; Castillo-Arellano et al. 2023; Vitale et al. 2021). These reviews focus on the complex interactions between CBD and various receptors, contributing to its therapeutic effects in disorders like epilepsy, anxiety, and depression. However, this manuscript expands upon these existing reviews by broadening the scope to include not only neuropsychiatric conditions but also multiple disease states and symptoms. By presenting a comprehensive approach to CBD receptor activation across various pathologies, this review offers a more integrated understanding of how CBD can influence a wide range of therapeutic outcomes, making it a valuable addition to the growing body of CBD literature for both researchers and clinicians.

**Fig. 1:**
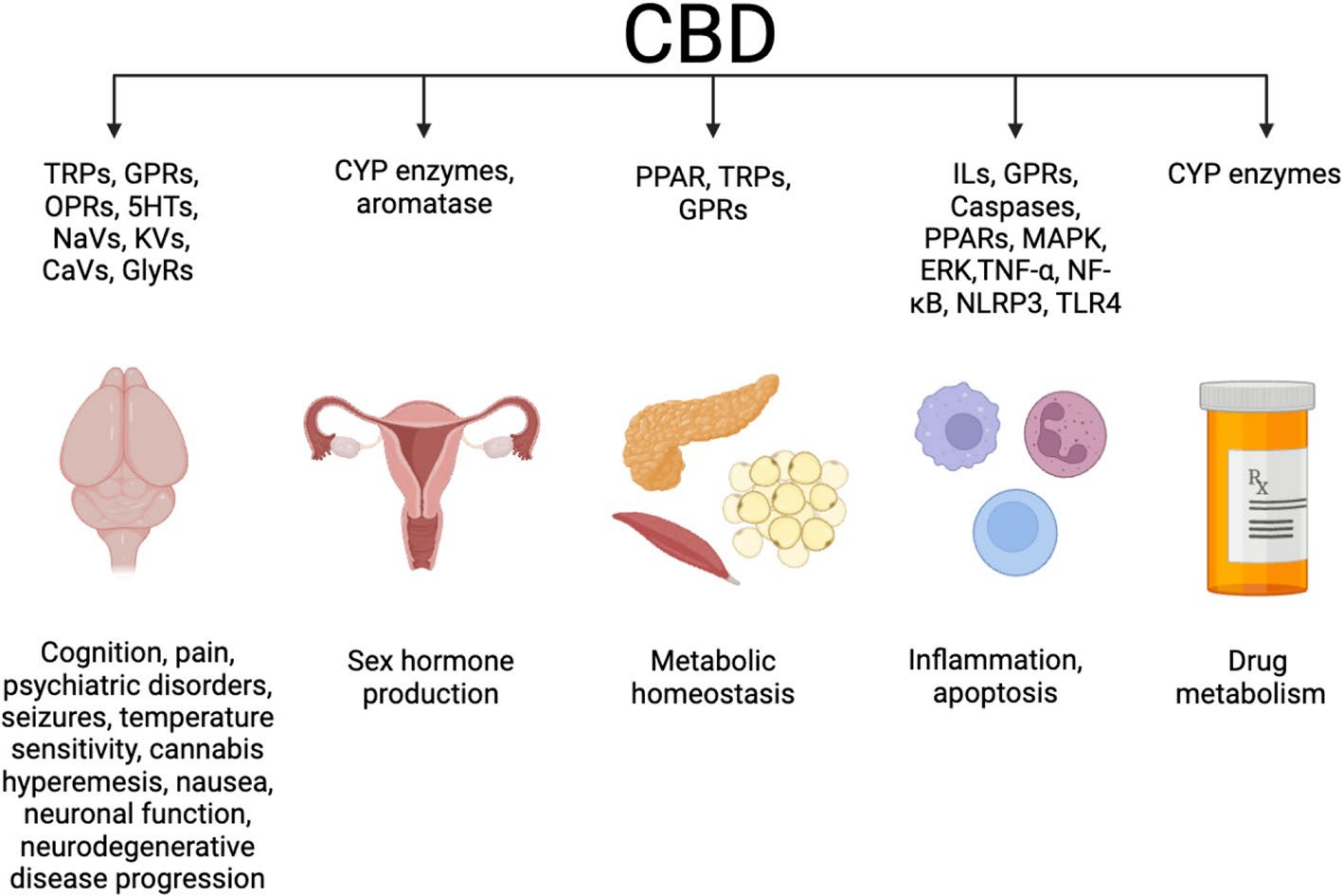
Graphical Summary. This graphical abstract summarizes the CBD pathways discussed, including the endocannabinoid system, ion channels, cytochrome P450 enzymes, those involved in energy homeostasis, inflammatory pathways, apoptotic pathways, and sex hormone regulation. Additionally, this figure introduces the biological processes discussed herein, including psychiatric disorders, seizure activity, nausea and vomiting, pain sensation, thermal regulation, neuronal signaling, neurodegenerative diseases, reproductive aging, drug metabolism, inflammation, sex hormone regulation, and energy homeostasis. Created with BioRender.com

With increasing recreational and medicinal consumption of CBD, it is pertinent to understand the drug activity. There are many modalities in which a ligand may interact with a receptor. As discussed by Miller and colleagues, receptors may be ion channel receptors, enzyme linked receptors, G-protein-coupled receptors, or nuclear receptors (Miller and Lappin 2023). Ligands can bind with receptors directly, either by agonizing or antagonizing (Miller and Lappin 2023). Ligands can bind at the active site, or allosterically away from the active site (Miller and Lappin 2023). There are multiple methods a ligand may decrease activity of a receptor, including antagonism, inhibition, competitive inhibition, or inverse agonism (Miller and Lappin 2023). Certain substances may also impact receptor activity indirectly by suppressing or modulating activity, altering the kinetics of a reaction, or altering the expression of a receptor or another ligand (Miller and Lappin 2023). CBD affects multiple receptors in all of these ways. CBD was initially hypothesized to signal solely though the endocannabinoid system, though investigations have revealed functional interactions with Transient Potential Vanilloid 1 (TRPV1) (Costa et al. 2004) and the 5-hydroxytryptamine (5HT) receptors, or serotonin receptors (Rock et al. 2012). A previous review by de Almeida and colleagues has highlighted CBD binding on a subset of G-protein-coupled receptors and ion channels (Almeida and Devi 2020). The goal of this review is to compile the literature regarding various biological processes in which CBD in involved and to build on prior discussions of CBD pathways (Fig. 1).

## Methods

### Search Strategy

I conducted a comprehensive literature search to compile evidence regarding cannabidiol (CBD) interactions with receptors, enzymes, and biological processes, organized by body systems. Searches were performed across PubMed, Web of Science, and Google Scholar for articles published through 2022. I included studies utilizing diverse methodologies, including cell culture, animal models, biochemical assays, and clinical research, to ensure a robust and holistic understanding of CBD receptor interactions. Search terms included combinations of the following keywords: "CBD," "cannabidiol," "receptors," "enzymes," "ion channels," "endocannabinoid system," "cytochrome P450," "inflammation," "hormones," and "neurotransmission." I screened titles and abstracts for relevance to the theme of CBD interactions with receptors and enzymes. Articles were included if they reported direct, indirect, or implied interactions of CBD with specific receptors, enzymes, or biological processes. Full-text articles available in English were retrieved for those meeting initial inclusion criteria.

### Data Extraction and Synthesis

Data from eligible studies were extracted, including receptor/enzyme name, methodology (e.g., cell culture, animal model, clinical trial), and key findings related to CBD interactions. To facilitate thematic analysis, extracted data were grouped by body system (e.g., nervous, endocrine, immune) and categorized by the specific receptor or enzyme involved. Findings were narratively synthesized to highlight patterns, gaps, and implications for health and disease.

### Quality Assessment

Given the narrative nature of this review, no formal quality assessment tools were applied. However, emphasis was placed on studies providing mechanistic insights, robust methodologies, or clinical relevance to ensure reliability in the synthesis.

### Reporting

Relevant findings are presented in both narrative and tabular formats, organized by body system, to enhance clarity and accessibility.

### CBD interacts with the endocannabinoid system

The endocannabinoid system encompasses a growing list of receptors that are bound by the endogenous cannabinoids, or endocannabinoids, 2-arachidonoyl glycerol (2-AG) (Sugiura et al. 1995) and arachidonoyl ethanolamide, or anandamide (AEA) (Felder et al. 1993). Though cannabis has been consumed for thousands of years (Bridgeman and Abazia 2017), it wasn’t until 1988 when Devane and colleagues characterized the first receptor than exogenous cannabinoids bound, called the cannabinoid receptor type 1 (CB_1_) (Devane et al. 1988). In 1992, the first endogenous cannabinoid that bound CB_1_ was isolated, called arachidonoylethanolamide, or anandamide (AEA) (Devane et al. 1992). In the last 30 years, the understanding of the endocannabinoid system has grown exponentially to include additional receptors, like transient receptors potential (TRP) channels (Costa et al. 2004), (Muller et al. 2019), and peroxisome proliferator activated receptors (PPAR) (O’Sullivan et al. 2009), (O’Sullivan 2016), as well as additional minor endocannabinoids like virodhamine (Porter et al. 2002) and 2-arachidonoyl glycerol ether (Hanuš et al. 2001). Further discussion on the function of the endocannabinoid system in the central nervous system was presented by Zou and Kumar (Zou and Kumar 2018) and Lu and Mackie (Lu and Mackie 2016).

**Fig. 2:**
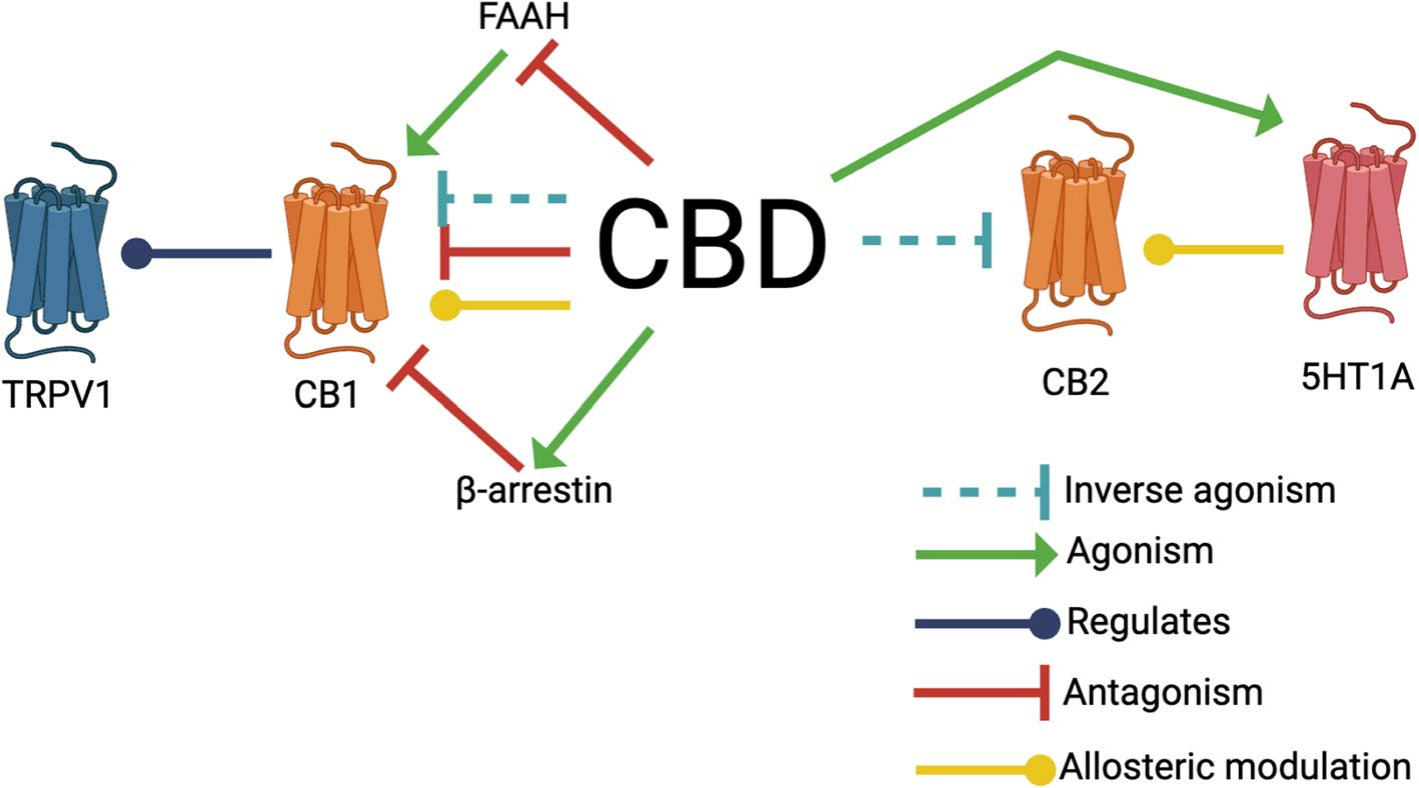
CBD interacts with the endocannabinoid system. CBD interacts with multiple regulators of the endocannabinoid system, including the endocannabinoid receptors CB_1_ and CB_2_. Additionally, CBD indirectly interacts with CB_1_ via the enzyme FAAH and β-arrestin. CBD also regulates TRPV1 via interactions with CB_1_ and inhibits 5HT_1A_ via interactions with CB_2_. Created with BioRender.com

CB_1_ and CB_2_ are activated by endogenous lipid-based retrograde neurotransmitters in the central and peripheral nervous system, including anandamide (AEA) (Felder et al. 1993) and 2-arachidonoylglyerol (2-AG) (Sugiura et al. 1995). CB_1_ is predominantly expressed in the central nervous system (Tissue expression of CNR1 - Summary - The Human Protein Atlas n.d) while CB_2_ is found in the peripheral nervous system and immune cells (Tissue expression of CNR2 - Summary - The Human Protein Atlas n.d), (Graham et al. 2010). CB_1_ and CB_2_ are G-protein-coupled receptors that confer intracellular signaling cascade activation when bound by ligands (Houston and Howlett 1998). CB_1_ and CB_2_ are activated by exogenous cannabinoid compounds such as THC (Shen and Thayer 1999). While CBD was initially theorized to activate CB_1_ and CB_2_ akin to the activation induced by THC, subsequent literature has debated this effect (McPartland et al. 2015) (Table 1, Fig. 2). Competitive binding affects downstream signaling by reducing receptor activation in a reversible manner, as higher concentrations of the endogenous ligand can outcompete the inhibitor and restore signaling. In contrast, non-competitive binding alters receptor function regardless of ligand concentration, often leading to partial or complete inhibition of downstream signaling by inducing conformational changes or disrupting signal transduction pathways. CBD has multiple effects on CB_1_ receptors, including inversely agonizing CB_1_ (Pertwee 2008) and serving as a negative allosteric modulator of CB_1_ (Laprairie et al. 2015), depending on the cellular context (Table 1, Fig. 2). For example, in HEK 293A cells that exogenously express CB_1_ receptors, and in a Huntington’s Disease model striatal cell line (ST*Hdh*^Q7/Q7^), application of CBD induced noncompetitive negative allosteric modulation of CB_1_ receptors with CB_1_ agonists (Laprairie et al. 2015) (Table 1, Fig. 2). CBD serves as an inverse agonist of CB_1_ at low levels in hCB_2_-CHO cells (Pertwee 2008) (Table 1, Fig. 2). Additionally, CBD alters the kinetics of internalization of CB_1_ receptors into the cell through β-arrestin recruitment (Table 1, Fig. 2) (Laprairie et al. 2015). CBD has an indirect effect on CB1 through antagonism of fatty acid amide hydrolase (FAAH) (Petrocellis et al. 2011), (Bisogno et al. 2001). FAAH breaks down the endocannabinoid anandamide (Kwilasz et al. 2014). By inhibiting FAAH, CBD can increase circulating anandamide levels (Hua et al. 2023), (Leweke et al. 2012), leading to prolonged activation of CB_1_ (Table 1, Fig. 2). CBD inversely activates CB_2_ (Thomas et al. 2007) (Table 1, Fig. 2). In a [ (Tissue expression of CNR2 - Summary - The Human Protein Atlas n.d) S]GTPγS binding assay using CHO cell membranes transfected with CB_2_ receptors (hCB_2_-CHO), 1 μM CBD showed a significantly lower K_B_ than K_i_, highlighting its function as an inverse agonist for CB_2_ (Thomas et al. 2007). Additionally, CBD induces a heterodimerization of CB_2_ with 5HT receptors (Pazos et al. 2013) (Table 1, Fig. 2). By regulating this heterodimer, CBD may be influential in neonatal hypoxic-ischemic brain damage (Pazos et al. 2013). Current work investigating the involvement of CBD and the endocannabinoid receptors is rapidly expanding, however there is still debate as to which of these effects are feasible at physiologic consumption levels of CBD.

**Table 1:** CBD interacts with the endocannabinoid system

Receptor/enzyme	Full receptor/enzyme name	Interaction	Reference(s)
FAAH	Fatty acid amide hydrolase	Antagonism	Bisogno et al. 2001; Petrocellis et al. 2011)
CB_1_	Cannabinoid receptor type 1	Inverse agonism, Negative allosteric modulator, Blocks	Navarro et al. 2020; Laprairie et al. 2015; Thomas et al. 2007)
CB_2_	Cannabinoid receptor type 2	Inverse agonism	Thomas et al. 2007)
Heterodimerization of CB_2_/5HT_1A_		Interaction	Pazos et al. 2013)
CB_1_R internalization		Affects the kinetics	Navarro et al. 2020)

### CBD interacts with ion channels

Ion channels control the flow of charged ions, such as K^+^, Na^+^, Ca^2+^, and Cl^−^ across the cell membrane. These ions regulate the cell membrane potential, which is critical in excitable tissues such as the brain, heart, and pancreas (Neher 1992). Cells within these tissues communicate through action potentials, chemical and electrical synapses, and gap junctions, all of which are mediated by the ion channels that control the membrane potential (Neher 1992). Altering the activity of these ion channels disrupts intercellular communication and can have downstream impacts on tissue function (Neher 1992).

### CBD impacts processes involved in psychiatric disorders

**Fig. 3:**
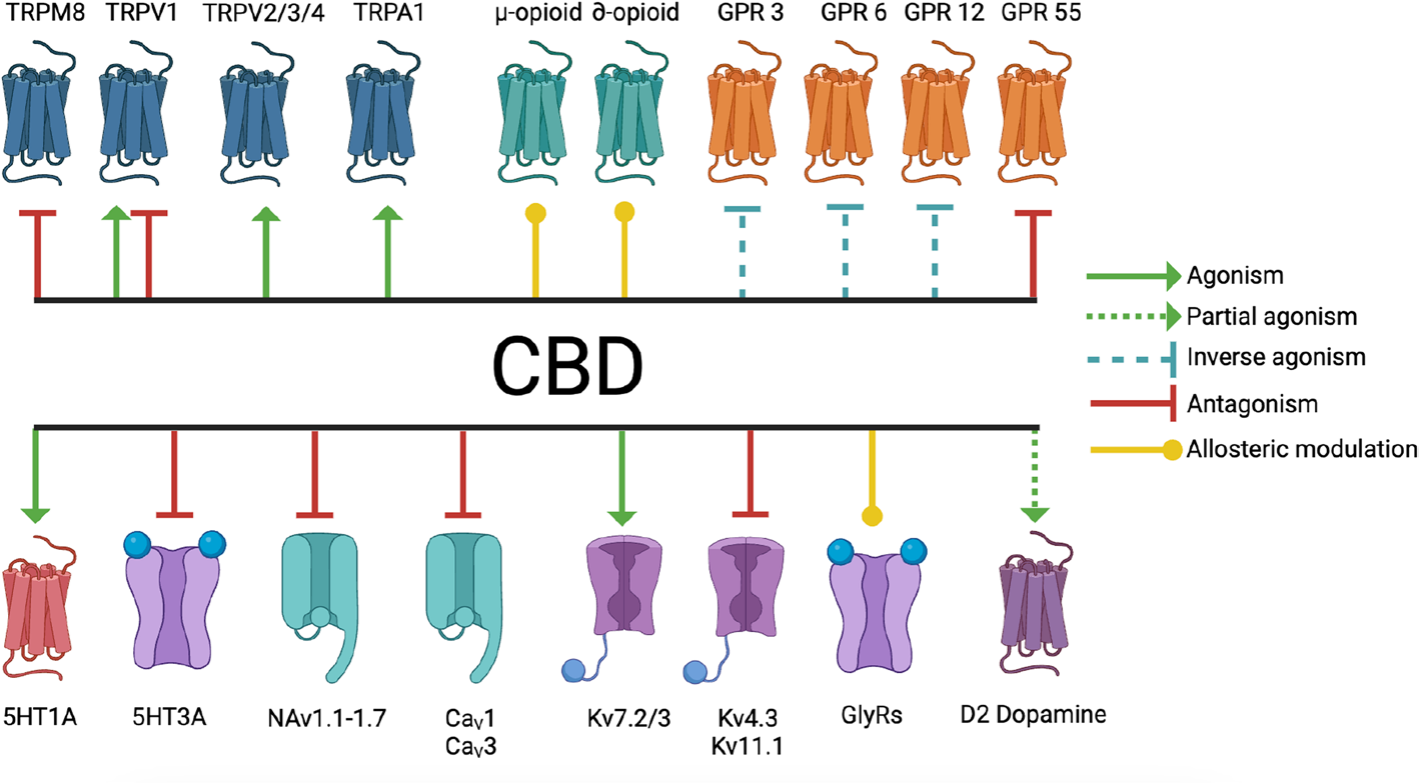
CBD interacts with ion channels and G-protein coupled receptors. CBD interacts with multiple ion channels, including the TRP channels TRPM8, TRPV1, TRPV2, TRPV3, TRPV4, and TRPA1, serotonin receptor 5HT_3A_, sodium channels Na_V_1.1–1.7, L-type calcium channels, voltage-gated potassium channels K_V_7.2, K_V_7.3, K_V_4.3, and K_V_11.1, and glycine receptors. CBD interacts with the G protein-coupled receptors GPR3, GPR6, GPR12, and GPR55, the μ-opioid and ∂-opioid receptors, and interacts with receptors who couple with G protein coupled receptors for downstream signaling cascades, including 5HT_1A_ serotonin receptors and D2 dopamine receptors. Created with BioRender.com

CBD activates and antagonizes processes that are implicated in the development or management of psychiatric disorders, specifically serotonin receptors (Rock et al. 2012; Xiong et al. 2011; Yang et al. 2010) and G-protein-coupled receptors 3, 6, 12 and 55 (Laun et al. 2019; Laun and Song 2017; Lauckner et al. 2008) (Table 2, Fig. 3). GPRs 3, 6, and 12 are also called cannabinoid-related orphan receptors, because of their reactivity to endogenous and exogenous cannabinoids (Laun et al. 2019). CBD activates 5HT_1A (_Rock et al. 2012), a G-protein-coupled receptor that is heavily expressed in the brain, gastrointestinal tract, endocrine tissues, kidney, and muscles, among other tissues (Tissue expression of HTR1A - Summary - The Human Protein Atlas n.d) (Table 2, Fig. 3). In the central nervous system, dysregulation of 5HT_1A_ signaling has harmful effects on cognition, mood and behavior disorders, depressive disorders, and panic disorders (Savitz et al. 2009; Akimova et al. 2009). CBD also indirectly decreases 5HT_1A_ expression (Jenny et al. 2009; Jenny et al. 2010) (Table 2, Fig. 3). Under CBD exposure, tryptophan, the precursor to 5HT_1A_, preferentially follows the IDO1/2 pathway to tryptophan catabolism instead of conversion to 5HT_1A (_Jenny et al. 2009; Jenny et al. 2010) (Table 2, Fig. 3)_._ CBD antagonizes another serotonin receptor, 5HT_3A (_Yang et al. 2010), which is expressed in the brain, digestive tract, pancreas, muscle, bone marrow, and lymphoid tissue (HTR3A protein expression summary - The Human Protein Atlas n.d) (Table 3, Fig. 3). Unlike the other 5HT receptors that couple to G-protein-coupled receptors, 5HT_3A_ is a ligand-gated ion channel (Rodriguez Araujo et al. 2020). In the central nervous system, 5HT_3A_ has been localized to pre- and post-synaptic nerve terminals in both excitatory and inhibitory neurons that release dopamine, cholecystokinin, and GABA (Engel et al. 2013). 5HT_3A_ dysregulation has similar effects to 5HT_1A_ in affecting mood disorders, as dysregulation of 5HT_3A_ is implicated in depression, bipolar disorder, and post-traumatic stress disorder (Jang et al. 2015; Bétry et al. 2011). CBD inversely activates GPR3 and GPR6 (Laun and Song 2017) (Table 2, Fig. 3). GPR3 is expressed in the brain, endocrine tissues, muscle, respiratory system, and digestive tract (Tissue expression of GPR3 - Summary - The Human Protein Atlas n.d), and GPR6 is expressed in the brain and endocrine tissues (Tissue expression of GPR6 - Summary - The Human Protein Atlas n.d). Activation of GPR3 and GPR6 impact behavior, where activation of GPR3 mediates behavioral changes in stress response (Valverde et al. 2009), and GPR6 alters instrumental learning by regulating cyclic adenosine monophosphate (cAMP) production in striatal spiny neurons (Oeckl et al. 2014). GPR3 activation also modulates cocaine reinforcement (Tourino et al. 2012), suggesting it may play a role in risk for addiction disorders. Together, these interactions implicate a potential effect of CBD consumption on the development, progression, or management of psychiatric disorders.

**Table 2:** CBD interacts with ion channels, enzymes, and G protein-coupled receptors

Ion channel/receptor/enzyme	Full receptor/ion channel/ enzyme name	Interaction	Reference(s)
**Serotonin related receptors and enzymes**			
5HT_1A_	5-hydroxytryptamine receptor 1A	Activates	Rock et al. 2012)
IDO1/2 to increase tryptophan catabolism	Indoleamine-pyrrole 2,3-dioxygenase	Activates	Jenny et al. 2009; Jenny et al. 2010)
5-HT_3A_	5-hydroxytryptamine receptor 3A	Antagonizes	Xiong et al. 2011; Yang et al. 2010)
**TRP receptors**			
TRPV1	Transient Receptor Potential Cation Channel Subfamily V Member 1	Activates, inhibits	Petrocellis et al. 2011; Anand et al. 2020)
TRPV2	Transient Receptor Potential Cation Channel Subfamily V member 2	Activates	Qin et al. 2008)
TRPV3	Transient Receptor Potential Cation Channel Subfamily V Member 3	Activates	Petrocellis et al. 2012)
TRPV4	Transient Receptor Potential Cation Channel Subfamily V Member 4	Activates	Petrocellis et al. 2012)
TRPA1	Transient Receptor Potential Cation Channel Subfamily A Member 1	Activates	Petrocellis et al. 2008)
TRPM8	Transient Receptor Potential Cation Channel Subfamily M Member 8	Antagonizes	Petrocellis et al. 2008)
**Other ion channels**			
K_V_7.2/3	Potassium voltage-gated channel subfamily KQT member 2 and 3	Activates	Zhang et al. 2022)
K_V_4.3	Potassium voltage-gated channel subfamily D member 3	Inhibits	Marois et al. 2020)
K_V_11.1	Potassium voltage-gated channel 11.1	Inhibits	Marois et al. 2020)
Na_V_1.1–1.7	Voltage-gated sodium channel	Inhibits	Marois et al. 2020), (Ghovanloo et al. 2018)
Cav1	L-type calcium channel	Inhibits	Marois et al. 2020), (Isaev et al. 2022)
Cav3	T type calcium channel	Inhibits	Ross et al. 2008)
GlyRs	Ligand-gated glycine receptors	Allosterically modulates	Ahrens et al. 2009)
**G protein-coupled receptors**			
GPR 3	G-protein-coupled receptor 3	Inversely activates	Laun et al. 2019), (Laun and Song 2017)
GPR 6	G-protein-coupled receptor 6	Inversely activates	Laun et al. 2019), (Laun and Song 2017)
GPR 12	G-protein-coupled receptor 12	Inversely activates	
GPR 55	G-protein-coupled receptor 55	Antagonizes	Akimova et al. 2009)
D2	D2 dopamine receptors	Partially activates	Seeman 2016)
μ-opioid	μ-opioid receptors	Allosterically modulates	Vaysse et al. 1987), (Kathmann et al. 2006)
∂-opioid	∂-opioid receptors	Allosterically modulates	Kathmann et al. 2006)

**Table 3:** CBD interacts with cytochrome p450 enzymes

Enzyme	Full enzyme name	Interaction	Reference(s)
CYP1A1	Cytochrome P450 1A1	Antagonism	Qian et al. 2019)
CYP1A2	Cytochrome P450 1A2	Antagonism	Qian et al. 2019)
CYP1B1	Cytochrome P450 1B1	Antagonism	Qian et al. 2019)
CYP2B6	Cytochrome P450 2B6	Antagonism	Doohan et al. 2021), (Qian et al. 2019)
CYP2C9	Cytochrome P450 2C9	Antagonism	Doohan et al. 2021)
CYP2C19	Cytochrome P450 2C19	Antagonism	Doohan et al. 2021), (Qian et al. 2019), (Jiang et al. 2013)
CYP2D6	Cytochrome P450 2D6	Antagonism	Yamaori et al. 2011), (Qian et al. 2019)
CYP2J2	Cytochrome P450 2J2	Antagonism	Qian et al. 2019)
CYP3A4	Cytochrome P450 3A4	Antagonism	Yamaori et al. 2011)
CYP3A5	Cytochrome P450 3A5	Antagonism	Yamaori et al. 2011)
CYP3A7	Cytochrome P450 3A7	Antagonism	Yamaori et al. 2011)

### CBD impacts processes that modulate seizure activity

Epidiolex is the single United States Food and Drug Administration (FDA) approved CBD medication, used to treat seizures associated with Lennox-Gastaut Syndrome, Dravet Syndrome, or Tuberous Sclerosis Complex patients over the age of 1^8^. CBD antagonizes GPR55 (Lauckner et al. 2008), a G protein-coupled receptor that is dysfunctional in epileptic patients (Rosenberg et al. 2023) (Table 2, Fig. 3). GPR55 is expressed in the brain, gastrointestinal tract, male reproductive tract, bone marrow, and lymphoid tissues (GPR55 protein expression summary - The Human Protein Atlas n.d). By indirectly blocking the malfunctioning GPR55 in epileptic patients, CBD can significantly reduce seizure episodes (Rosenberg et al. 2023). Additional ion channels involved in seizure activity modulation are K_V_7.2 and K_V_7.3 (Miceli et al. 2015), which CBD activates (Zhang et al. 2022) (Table 2, Fig. 3). CBD inhibits K_V_4.3 (Marois et al. 2020), a potassium channel whose dysregulation via genetic mutation causes epilepsy (Smets et al. 2015) and K_V_11.1 (Marois et al. 2020), a potassium channel whose dysregulation induces seizures (Keller et al. 2009). CBD inhibits voltage-gated sodium channels, Na_V_1.1–1.7 (Ghovanloo et al. 2018) (Table 2, Fig. 3). Genetic disruptions in Na_V_ channels induce seizure activity due to alterations in action potential propagation, as discussed by Menezes and colleagues (Menezes et al. 2020). In genetic seizure disorders, pharmaceutical Na_V_ channel agonists or antagonists like carbamazepine and lamotrigine can be therapeutic in mitigating seizure activity by restabilizing action potential propagation (Catterall 2014). Because CBD is an effective seizure-reducing medication, and it is federally approved for treatment of childhood seizures (Abu-Sawwa et al. 2020), the mechanistic understanding of how CBD can mitigate seizure activity is an active area of investigation.

### CBD impacts receptors that regulate temperature sensitivity

CBD may impact temperature sensation by agonizing or antagonizing transient potential receptors, or TRPs (Table 2, Fig. 3) (Petrocellis et al. 2011; Anand et al. 2020; Petrocellis et al. 2012). CBD activates five TRP channels, including TRPV1, TRPV2, TRPV3, TRPV4, and TRPA1, and antagonizes TRPM8 (Petrocellis et al. 2011; Anand et al. 2020; Qin et al. 2008; Petrocellis et al. 2012; Petrocellis et al. 2008) (Table 2, Fig. 3). TRPV1 and TRPV2 sense high thermal stimuli, including stimuli above 42 °C and above 52 °C, respectively (Samanta et al. 2018). TRPM8 and TRPA1 sense low thermal stimuli, including temperatures 25–34 °C and below 17 °C, respectively (Samanta et al. 2018). TRPV1 is expressed in the brain, liver, gallbladder, pancreas, muscle, and reproductive tissues, TRPV2 is expressed in the brain, endocrine tissues, respiratory system, gastrointestinal tract, liver, gallbladder, and pancreas, among other tissues, TRPM8 is expressed in the liver, gallbladder, and male reproductive tissues, and TRPA1 is expressed in the gastrointestinal tract, liver, gallbladder, kidney, bladder (TRPA1 protein expression summary - The Human Protein Atlas n.d; Tissue expression of TRPV1 - Summary - The Human Protein Atlas n.d; TRPV2 protein expression summary - The Human Protein Atlas n.d; TRPM8 protein expression summary - The Human Protein Atlas n.d). TRPV3 is expressed predominantly in the gastrointestinal tract and skin, with lower expression levels in the muscle, reproductive tissues, brain, and endocrine tissues (TRPV3 protein expression summary - The Human Protein Atlas n.d). TRPV4 is expressed in the brain, endocrine tissues, gastrointestinal tract, pancreas, reproductive tissues, and muscle tissues, among others (TRPV4 protein expression summary - The Human Protein Atlas n.d). Dysregulation of TRP channels can alter thermal pain sensation, as discussed by Cortright and colleagues (Cortright et al. 2007). Exposure to TRP agonists can increase thermal pain sensitivity (Cortright et al. 2007). In fact, intrauterine CBD exposure increases sensitivity to thermal pain in adult male offspring in a TRPV1 dependent manner (Swenson et al. 2023). The agonism of these six receptors by CBD signals that CBD consumption could potentially mediate thermal sensitivity.

### CBD content may modulate cannabis-induced hyperemesis

Following prolonged consumption of cannabis, some patients experience severe refractory nausea and vomiting called cannabis hyperemesis (Perisetti et al. 2020). The mechanism of cannabis hyperemesis is yet to be elucidated, though one working mechanistic theory is that prolonged THC exposure induces TRPV1 hypersensitization in the enteric and vagal neurons (Sharkey 2022). Interestingly, many patients with cannabis hyperemesis report relief following a hot shower (Perisetti et al. 2020), suggesting potential involvement of TRP receptors. Because TRPV1 is responsive to high heat (Samanta et al. 2018), and because it is expressed in the area postrema of the medulla, along gastric enteric and vagal nerves, and on cutaneous receptors in the dermis and epidermis (Tissue expression of TRPV1 - Summary - The Human Protein Atlas n.d), researchers postulate that repetitive TRPV1 activation may cause vomiting from overexcitation, or relief from the vomiting under acute activation of the hot shower. As CBD activates TRPV1, its role in inducing or mediating cannabis hyperemesis is theoretical and a key point for future investigations.

### CBD impacts processes that regulate nausea and vomiting

CBD has gained traction recently as an anti-emetic, or nausea-reducing, medication. One potential mechanism by which CBD may inhibit nausea and vomiting is through antagonism of 5HT_3A_ receptors (Yang et al. 2010; Theriot et al., n.d) (Table 2, Fig. 3). In the enteric nervous system that lines the gastrointestinal tract, 5HT_3A_ receptor antagonists inhibit the gastrointestinal activity in nausea and vomiting (Browning 2015). Additionally, it is theorized that 5HT_3A_ antagonism in the area postrema in the brainstem, known as the vomiting center, decreases the nausea and vomiting response in small mammals (Higgins et al. 1989). In rodent studies, CBD suppresses 0.1% saccharin solution induced vomiting in Asian house shrews (S. Murinus) and conditioned gaping (a measure of rodent nausea) in rats due to indirect agonism of 5HT_1A_ somatodendritic autoreceptors in the dorsal raphe nucleus (Rock et al. 2012). CBD is increasingly consumed as an anti-emetic medication, particularly by chemotherapy patients, pregnant patients, and migraine patients (O’Brien 2022; Baron 2018). As CBD inhibits CYP 450 enzymes that metabolize pharmaceuticals (Smith and Gruber 2023), (Doohan et al. 2021), (Qian et al. 2019) understanding the impacts of CBD usage in chemotherapy is critical. Additionally, understanding the potential impact of CBD on fetal development, and the impact of co-consumption of CBD with migraine medications, would help inform these patients to the safety, risks, or drug-drug interactions that are possible with CBD. As CBD does not cause many of the side effects that accompany other nausea medications, like constipation and headache (Tincello and Johnstone 1996), it is a promising area of clinical investigation.

### CBD impacts processes that regulate pain sensation

**Fig. 4:**
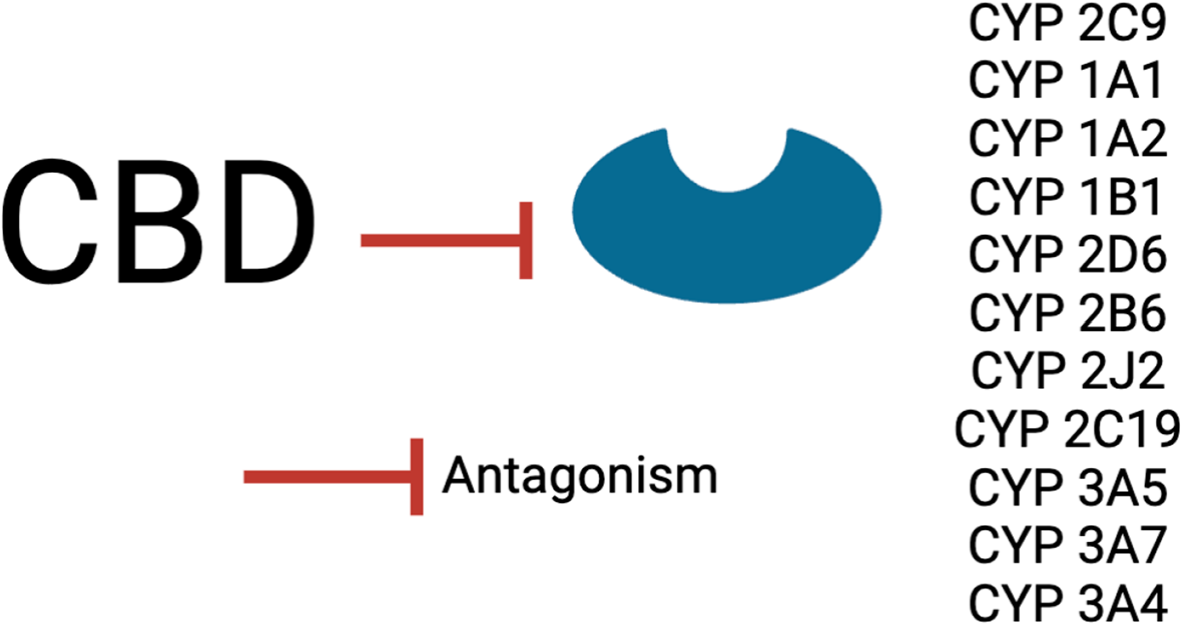
CBD interacts with cytochrome p450 enzymes. CBD competitively inhibits CYP enzymes, including CYPs 2C9, 1A1, 1A2, 1B1, 2D6, 2B6, 2J2, 2C19, and antagonizes CYPs 3A5, 3A7, and 3A4. Created with BioRender.com

CBD impacts multiple processes that regulate pain sensation, including ion channels that sense painful stimuli, opioid receptors, and enzymes which regulate the breakdown of pain medications. CBD activates five TRP channels, including TRPV1, TRPV2, TRPV3, TRPV4, and TRPA1, and antagonizes TRPM8 (Table 2, Fig. 3) (Petrocellis et al. 2011; Anand et al. 2020; Qin et al. 2008; Petrocellis et al. 2012; Petrocellis et al. 2008). TRPV1 and TRPA1 antagonists are under current clinical trials as medications to reduce inflammatory, neuropathic, and visceral pain conditions (Gunthorpe and Chizh 2009; Giorgi et al. 2019). In addition to the thermal pain sensation mediated by TRP channels, CBD also impacts receptors that mediate nociceptive pain. CBD is an allosteric modulator of both μ-opioid receptors and ∂-opioid receptors at high concentrations, altering the efficacy of which opioid agonists bind or dissociate from the receptors (Vaysse et al. 1987; Kathmann et al. 2006) (Table 2, Fig. 3). Though CBD does allosterically modulate these opioid receptors, the authors who published on this interaction discuss how it is unlikely that CBD would induce these interactions at physiologically relevant levels (Kathmann et al. 2006). Kathmann discusses how the half maximal effective concentration (EC_50_) needed for CBD to interact with opioid receptors is likely 100 times higher than what can be consumed in a standard oral dose of CBD (Kathmann et al. 2006). This discussion was later supported by human pharmacokinetic studies which show plasma CBD metabolite levels following various levels of CBD ingestion, as discussed by Ujváry and colleagues (Ujváry and Hanuš 2016). Additionally, serotonin receptors such as 5HT_1A_ regulate neuropathic pain conditions such as migraine and fibromyalgia (Leone et al. 1998; Tour et al. 2022). By activating 5HT_1A_, CBD inhibits paclitaxel-induced neuropathic pain (Ward et al. 2014). In the context of pain management, CBD may also impact the effectiveness of standard medications, including codeine, hydrocodone, oxycodone, fentanyl, meperidine, methadone, buprenorphine, and tramadol, all of which are metabolized by the cytochrome p450 (CYP) enzyme group (Table 3, Fig. 4) (Doohan et al. 2021; Interactions Between Cannabinoids and Cytochrome P450-Metabolized Drugs - Full Text View - ClinicalTrials.gov n.d). By antagonizing or competitively inhibiting the CYP enzymes (Doohan et al. 2021; Qian et al. 2019; Yamaori et al. 2011 Yamaori et al. 2011), it is possible that CBD co-consumption with narcotics will increase the narcotic half-life in the system, increasing pain management, but also increasing risk of overdose. In clinical studies investigating the beneficial role of CBD in pain management, co-consumption of CBD with opioids allowed patients to decrease opioid dose while maintaining effective levels of pain relief, though it is only speculated that this could be through a CYP enzyme inhibition mechanism (Capano et al. 2020). CBD antagonizes CYP2D6 (Qian et al. 2019; Yamaori et al. 2011), which metabolizes opioids (Yamaori et al. 2011) (Table 3, Fig. 4). By inhibiting this enzyme, CBD may hinder the breakdown of opioids and alter the half-life of the opioids in the bloodstream. CBD may also alter the metabolism of painkillers ketamine and methadone via antagonizing CYP2B6 (Doohan et al. 2021; Qian et al. 2019) (Table 3, Fig. 4). CBD inversely activates GPR3 (Laun et al. 2019), and GPR3 knockout mice show increased pain sensitivity and reduced response to morphine, highlighting the role of GPR3 in pain sensation (Ruiz-Medina et al. 2011) (Table 2, Fig. 3). CBD is an allosteric modulator of GlyRs (Ahrens et al. 2009), which mediate pain processing and pain hypersensitivity (Moraga-Cid et al. 2020) (Table 2, Fig. 3). CBD also activates K_V_7.2/3 (Zhang et al. 2022), whose activation in sensory nociceptive neurons mediates how Aδ peripheral nerves respond to noxious heat as discussed by Brown and Colleagues (Brown and Passmore 2009) (Table 2, Fig. 3). Together, these data implicate how consumption of CBD can alter pain sensation, which can have long-lasting impacts on pain tolerance, pain sensitivity, and consumption of pain-reducing medications.

### CBD impacts neuronal function

CBD consumption may mediate neuronal function by agonizing or antagonizing multiple ion channels that maintain neuronal membrane potential, including potassium channels, sodium channels, and serotonin receptors, as well as G-protein-coupled receptors. CBD interacts with multiple ion channels, all of which have the potential to mediate neuronal signaling by altering neuronal membrane potential. CBD activates voltage-gated potassium channels K_V_7.2 and K_V_7.3 (Table 2, Fig. 3) (Zhang et al. 2022). As discussed by Brown and colleagues, K_V_7.2 and K_V_7.3 are expressed in the nervous system and regulate neuronal excitability (Brown and Passmore 2009). In sympathetic neurons, K_V_7.2/3 activity mediates repetitive discharges and conversion from phasic to tonic firing, and in hippocampal pyramidal neurons, K_V_7.2/3 activity mediates repetitive discharges of the neuron (Brown and Passmore 2009). By agonizing these ion channels and altering these repetitive discharges, CBD could be altering neuronal communication. CBD also inhibits Na_V_1.1–1.7 channels and L-type and T-type calcium channels (Ghovanloo et al. 2018; Isaev et al. 2022; Ross et al. 2008; Ghovanloo and Ruben 2022; Ali et al. 2015) all of which regulate action potential propagation and subsequent neuron communication (Grider et al. 2023) (Table 2, Fig. 3). In fact, fetal CBD exposure reduces the excitability of the prefrontal cortex and cognition in females (Swenson et al. 2023). By disrupting sodium and potassium channels which regulate neuronal communication, CBD may have lasting impacts on neuronal network structure and subsequent function.

CBD inversely activates GPRs 3, 6, and 12 (Table 2, Fig. 3) (Laun et al. 2019). The exact mechanism of these receptors is yet to be classified, but they have proposed mechanisms based on structural similarity to other receptors (Laun and Song 2017). GPRs 3, 6, and 12 are structurally similar to cannabinoid receptors, and the involvement of CBD with these receptors has been under recent investigation (Laun and Song 2017). GPR12 is expressed in the brain, eye, and gastrointestinal tract (GPR12 protein expression summary - The Human Protein Atlas n.d). All three receptors impact neurological functioning, where activation of GPR3, GPR6, and GPR12 mediates neurite outgrowth (Tanaka et al. 2007) and activation of GPR3, GPR6 and GPR12 regulates neuronal survival (Tanaka et al. 2014; Full article: Towards a better understanding of the cannabinoid-related orphan receptors GPR3, GPR6, and GPR12 n.d). CBD is an allosteric modulator of GlyRs (Ahrens et al. 2009), which are ligand-gated ion channels that regulate motor coordination, respiratory control, and muscle tone by controlling action potential activity (Moraga-Cid et al. 2020) (Table 2, Fig. 3).

CBD activates 5HT_1A_, 5HT_3A_, and D2 dopamine receptors (Rock et al. 2012; Yang et al. 2010; Seeman 2016), which mediate neurotransmitter release and neuronal communication (Altieri et al. 2012; Fields et al. 1990; Bhatt et al. 2021; Wu and Hablitz 2005). 5HT_1A_ and 5HT_3A_ play crucial roles in mediating neuronal signaling (Altieri et al. 2012; Bhatt et al. 2021). The agonism of 5HT_1A_ receptors hyperpolarizes the neuron, leading to a decrease in action potential propagation (Sprouse and Aghajanian 1986). As action potential propagation increases neurite outgrowth (Fields et al. 1990), CBD may hinder neurite outgrowth and subsequent neuronal connections. 5HT_3A_ receptor antagonism is under current investigation for its therapeutic effects in depression models (Bhatt et al. 2021). CBD antagonizes 5HT_3A_ (Yang et al. 2010)_,_ meaning CBD may play a role in depressive disorders. CBD is a partial agonist of D2 dopamine receptors (Table 2, Fig. 3) (Seeman 2016). As agonism of D2 dopamine receptors increases neurite outgrowth (Todd 1992), CBD exposure may subsequently increase outgrowth.

These receptors, including the 5HT_1A_, 5HT_3A_, TRP channels, D2 dopamine receptors, K_V_7.2, K_V_7.3, Na_V_1.1–1.7, and L-type and T-type calcium channels, may have synergistic or oppositional effects upon CBD binding. The receptors and channels mediate many components of action potential propagation, including the influx of sodium ions during depolarization and the efflux of potassium during repolarization and hyperpolarization. Some receptors and channels have the ability to mediate activity of other receptors or channels often through the alteration of the membrane potential. Sodium channels, including Na_V_1.1–1.7, are responsible for the inward flux of sodium ions that depolarize a neuron (Eijkelkamp et al. 2012). Inhibition of these channels by CBD would decrease neuronal excitability by preventing membrane voltage from raising above baseline. 5HT_1A_ agonism opens potassium channels (allowing potassium influx and repolarization/hyperpolarization) and closing calcium channels (hindering depolarization) (Ehrengruber et al. 1997; Penington et al. 1991; Albert and Vahid-Ansari 2019). By agonizing 5HT_1A_ receptors (Rock et al. 2012), CBD may additionally decrease action potential propagation by hyperpolarizing the cell (Albert and Vahid-Ansari 2019). In some neuronal subtypes, calcium channels serve as upstream moderators of action potential propagation by dictating membrane potential (Iosub et al. 2015). For example, in the calcium-induced calcium release in inner hair cells, L type calcium channel activity induces the opening of potassium channels during repolarization (Iosub et al. 2015). Similarly, agonism of T type calcium channels induces depolarization when the neurons are in a hyperpolarized state (Cain and Snutch 2010). T type calcium channels are also involved in the repolarization and hyperpolarization of membrane potential, as they activate voltage-gated potassium channels that allow an outward flux of positively charged potassium ions (Cain and Snutch 2010). By inhibiting these calcium channels, CBD may further decrease depolarization. Voltage-gated potassium channels, including K_V_7.2 and 7.3, respond to changes in membrane potential to allow the outward flux of potassium ions during the latter stage of the action potential (Estacion et al. 2023). This outward flux of potassium pushes the membrane voltage back to baseline, and later to hyperpolarization (Estacion et al. 2023). By agonizing these channels, CBD may additionally decrease action potential propagation. In opposition, many of the TRP receptors induce depolarization upon activation (Gees et al. 2010), including when activated specifically by CBD (Kowalski et al. 2020). Similarly, D2 dopamine receptor agonism induces depolarization (Wu and Hablitz 2005).

### CBD interacts with receptors that affect neurodegenerative disease progression and symptom management

CBD is under investigation for its impact on treating symptoms of neurodegenerative diseases, such as Parkinson’s disease and Alzheimer's disease (Bhunia et al. 2022). Neurodegeneration is a complex process regulated by many receptors, some of which are bound by CBD. As discussed by Bhunia and colleagues, CB_1_R, CB_2_R, PPARγ, 5HT_1A_, A_2A_-R, and TRPV1 all have neuroprotective effects (Table 2, Fig. 3) (Bhunia et al. 2022). In addition to these receptors, CBD also inversely activates GPR3 and GPR12 (Table 2, Fig. 3) (Laun et al. 2019; Laun and Song 2017). GPR3 and GPR6 have shown an impact on Alzheimer’s disease progression by regulating amyloid beta production (Huang et al. 2022), and activation of GPR6 modulates Parkinson’s disease progression by regulating striatal dopamine production (Brice et al. 2021). CBD is currently under investigation in clinical trials for symptom management in Parkinson’s Disease and Alzheimer’s disease, and in disease progression using animal model studies though the mechanism behind potential benefits has not yet been defined (Chagas et al. 2014; Chesworth et al. 2022; Hao and Feng 2021; McManus et al. 2021; Almeida et al. 2023; Faria et al. 2020; Zhang et al. 2022).

### CBD antagonizes CYP enzymes which metabolize pharmaceuticals

CBD may impact pharmacologic drug metabolism by antagonizing CYP enzymes (Doohan et al. 2021; Qian et al. 2019; Yamaori et al. 2011 Yamaori et al. 2011) (Table 3, Fig. 4), the largest regulator of drug metabolism (Zhao et al. 2021). CYP enzymes are predominantly expressed in the liver, but are also present in the kidney, placenta, adrenal gland, gastrointestinal tract, and skin (Zhao et al. 2021). Additionally, CYP enzymes are critical to produce cholesterol, steroids, prostacyclins, and thromboxane A_2 (_Rendic and Guengerich 2018). CBD has two primary effects on CYP enzymes that suppress CYP enzyme activity: antagonism and competitive inhibition (Smith and Gruber 2023; Doohan et al. 2021; Qian et al. 2019; Yamaori et al. 2011 Yamaori et al. 2011) (Table 3, Fig. 4). CBD competitively inhibits CYPs 3A4, 3A7, and 3A5 (Doohan et al. 2021; Yamaori et al. 2011) (Table 3, Fig. 4). CYP3A4 breaks down small foreign organic molecules (xenobiotics) that are common prescription medications, such as clarithromycin, erythromycin, diltiazem, itraconazole, ketoconazole, ritonavir, and verapamil (Sweeney and Bromilow 2006). By competitively inhibiting CYP3A4 (Smith and Gruber 2023), CBD can interfere with drug metabolism, increasing the half-life of the drug. During fetal liver development, CYP3A7 is the predominant CYP, while CYP3A4 takes over during postnatal development (Li and Lampe 2019). CYP3A7 hydroxylates testosterone and dehydroepiandrosterone 3-sulphate, which is involved in the formation of estradiol during pregnancy (CYP3A7 Gene - Cytochrome P450 Family 3 Subfamily A Member 7 n.d). By inhibiting CYP3A7 (Yamaori et al. 2011), CBD may have impacts on estradiol creation or maintenance. Unlike the majority of CYP enzymes that function in the liver, CYP3A5 metabolizes endogenous steroids and xenobiotics in extrahepatic tissues, including the lung, kidney, prostrate, breast and leukocytes (Lamba et al. 2002). This activity highlights potential risks of co-consuming CBD with common pharmaceutical or recreational medications as CBD may alter drug metabolism and subsequent activity.

CBD antagonizes CYP2C9, CYP1A1, CYP1A2, CYP1B1, CYP2D6, CYP2B6, and CYP2J2 (Smith and Gruber 2023; Doohan et al. 2021; Qian et al. 2019; Yamaori et al. 2011 Yamaori et al. 2011) (Table 3, Fig. 4). CYP2C9 is the predominant metabolizer of the blood clot prevention medication Warfarin (Dean and Warfarin Therapy and VKORC1 and CYP Genotype. In: Pratt VM, Scott SA, Pirmohamed M, Esquivel B, Kattman BL, Malheiro AJ, eds. Medical Genetics Summaries. National Center for Biotechnology Information (US) 2012). By antagonizing CYP2C9, CBD impairs the degradation of Warfarin, impacting blood clotting (Grayson et al. 2017; Cortopassi 2020; Hsu and Painter 2020). CYP1A1 is critical in cancer regulation because it metabolizes carcinogens into epoxide intermediates which are less detrimental (Androutsopoulos et al. 2009). CBD increases CYP1A1 expression in a Hep2G cell line and antagonizes the enzyme activity (Qian et al. 2019; Yamaori et al. 2015). CYP1A2 metabolizes endogenous compounds including retinols, melatonin, steroids, uroporphyrinogen, and arachidonic acid, as well as recreational and pharmaceutical drugs including phenacetin, caffeine, clozapam, tacrine, propranolol, and mexiletine (Zhou et al. 2009). CYP1A2 also metabolizes precarcinogens, including aflatoxins, mycotoxins, and nitrosamines (Zhou et al. 2009). By antagonizing CYP1A2 (Qian et al. 2019), CBD exposure could alter the breakdown of these substances. CYP1B1 metabolizes exogenous compounds akin to other CYP enzymes, while also metabolizing endogenous compounds such as estrogen, arachidonic acid, melatonin, and retinoids (Li et al. 2017). CYP2D6 metabolizes pharmaceutical medications including antidepressants, neuroleptics, some antiarrhythmics, lipophilic β-adrenoceptor blockers and opioids (Bertilsson et al. 2002). CBD inhibits CYP2C19 (Doohan et al. 2021; Qian et al. 2019), an enzyme that metabolizes multiple pharmaceutical drugs including citalopram, clomipramine, clopidrogrel, diazepam, omeprazole (Jiang et al. 2013). CYP2B6 is responsible for metabolizing pharmaceuticals including artemisinin, bupropion, cyclophosphamide, efavirenz, ketamine, and methadone (Zanger and Klein 2013). CYP2J2 metabolizes many pharmaceuticals, including antihistamines (terfenadine, ebastine, and astemizole), anticancer agents (doxorubicin and tamoxifen), and immunosuppressants (cyclosporine) (Solanki et al. 2018). Combined, these interactions demonstrate CBD is not inert, and CBD consumption can alter metabolism of many substances. Because of these interactions, healthcare providers and pharmacists should inquire about patient CBD consumption.

### CBD impacts the breakdown of exogenous cannabinoids

CBD hinders the breakdown of tetrahydrocannabinol (Zamarripa et al. 2023), or THC, the primary psychoactive component of marijuana by inhibiting CYP2C9, CYP2D6, and CYP3A4 (Doohan et al. 2021; Qian et al. 2019; Yamaori et al. 2011; Ng et al. 2023). CBD is the second most common cannabinoid included in marijuana products, followed by minor cannabinoids like cannabinol, cannabichromene, cannabigerol, cannabinolic acid, and cannabidivarin, among others (Walsh et al. 2021). CBD antagonizes CYP2C9 and CYP2C19 (Doohan et al. 2021; Qian et al. 2019; Jiang et al. 2013), which are the predominant metabolizers of exogenous cannabinoids (Bland et al. 2005) (Table 3, Fig. 4). Through this mechanism, CBD exposure could slow the metabolism of other exogenous cannabinoids, increasing their half-life and therefore increasing the length of symptomatology from the psychoactive components.

### CBD may alter the regulation of sex hormones

**Fig. 5:**
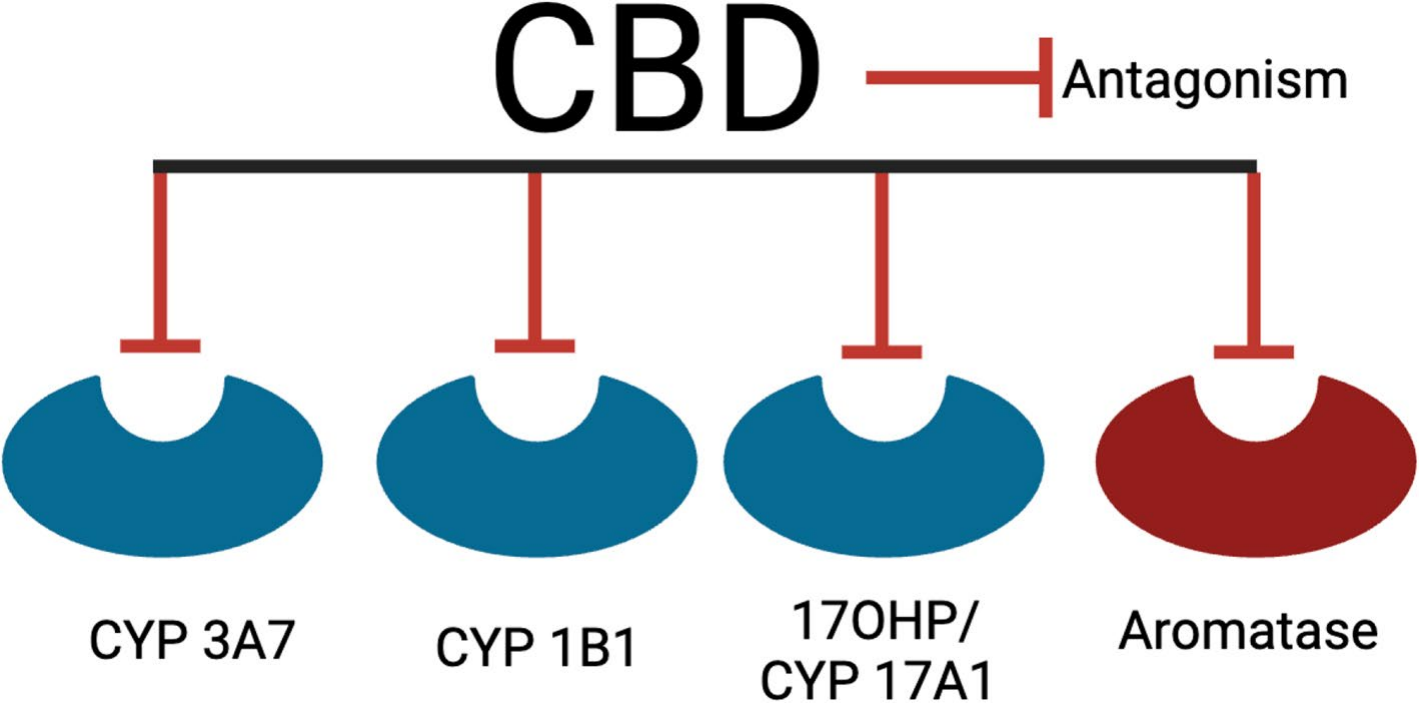
CBD may alter the regulation of sex hormones. CBD antagonizes multiple processes that regulate sex hormones, including CYP enzymes 3A7, 1B1, and 17A1, and aromatase. Created with BioRender.com

By interacting with multiple regulators of sex hormone production or metabolism, CBD may alter sex hormone production or levels. CBD antagonizes aromatase (Almada et al. 2020), an enzyme that converts testosterone to estrogen (Brodie et al. 1999) (Table 4, Fig. 5). Inhibition of aromatase during fetal development can be harmful, as it decreases the production of estrogens that are required for pregnancy maintenance and offspring sexual development (Tiboni and Ponzano 2016). Because of this, many aromatase inhibitor pharmaceuticals are contraindicated during pregnancy (Tiboni and Ponzano 2016). CBD antagonizes progesterone 17 hydroxylase (Watanabe et al. 2005), which hydroxylates pregnenolone and progesterone (precursors to aldosterone), to form 17-hydroxypregnenolone and 17-hydroxyprogesterone (precursors to cortisol) (Chormanski and Muzio 2023) (Table 4, Fig. 5). CYP3A7 hydroxylates testosterone and dehydroepiandrosterone 3-sulphate, a critical process in the production of estriol during pregnancy (CYP3A7 Gene - Cytochrome P450 Family 3 Subfamily A Member 7 n.d). By inhibiting CYP3A7 (Yamaori et al. 2011), CBD may have impacts on estriol creation or maintenance. CBD antagonizes CYP1B1 (Qian et al. 2019) (Table 4, Fig. 5). CYP1B1 metabolizes exogenous compounds akin to other CYP enzymes, while also metabolizing endogenous compounds such as estrogen, arachidonic acid, melatonin, and retinoids (Li et al. 2017). CBD inversely activates GPR3 and GPR12 (Table 2, Fig. 5) (Laun et al. 2019). GPR3 and GPR12 have roles in female reproduction through ovarian aging, where both receptors maintain meiotic arrest of oocytes and premature ovarian aging (Hinckley et al. 2005). By antagonizing or inhibiting this complex of processes, CBD could be altering sex hormone pathways.

**Table 4:** CBD may alter the regulation of sex hormones

Enzyme	Full enzyme name	Interaction	Reference(s)
Aromatase	Estrogen synthetase/synthase	Antagonism	Almada et al. 2020)
17OHP/CYP17A1	Progesterone 17-hydroxylase	Antagonism	Watanabe et al. 2005)
CYP3A7	Cytochrome P450 3A7	Inhibition	Yamaori et al. 2011)
CYP1B1	Cytochrome P450 1B1	Antagonism	Yamaori et al. 2010)

### CBD impacts processes that mediate metabolic homeostasis

**Fig. 6:**
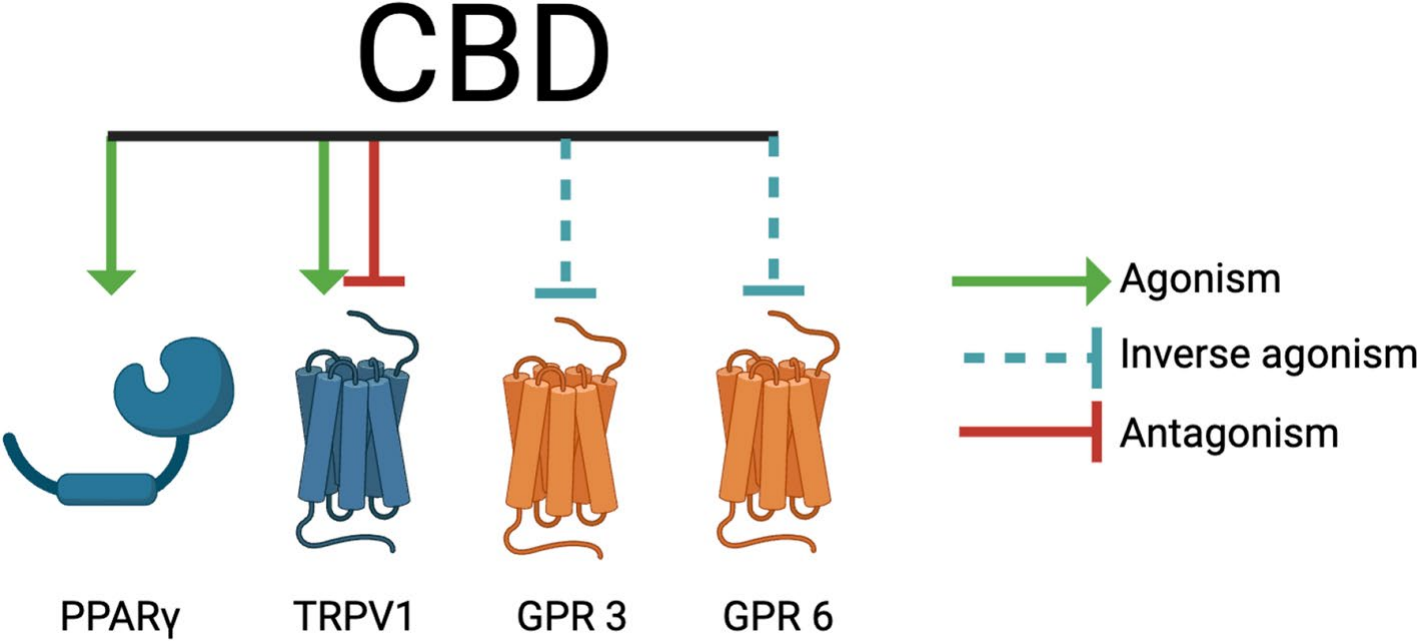
CBD interacts with regulators of energy homeostasis. CBD impacts regulators of energy homeostasis, including agonizing PPARγ, inversely agonizing GPR3 and GPR6, and agonizing and inhibiting TRPV1. Created with BioRender.com

CBD affects energy homeostasis and metabolism via multiple mechanisms. CBD accumulates in fat, muscle, and liver following consumption, however, females showed higher accumulation in the muscle and liver compared to males (Child and Tallon 2022). As discussed by Wiciński and colleagues, CBD impacts multiple metabolic processes, including in maintaining glucose homeostasis, regulating adipose tissue insulin sensitivity, maintaining low density lipid (LDL) and high density lipid (HDL) profiles, hypertension, and in the treatment of metabolic syndrome in clinical studies (Wiciński et al. 2023). CBD activates PPARγ (O’Sullivan 2016) (Table 5, Fig. 6). PPARγ activation promotes fatty acid uptake, triglyceride formation and storage in lipid droplets (Montaigne et al.^ 2021^). This activation in turn increases insulin sensitivity and glucose metabolism (PPARδ regulates glucose metabolism and insulin sensitivity | PNAS n.d). As such, CBD may impact insulin sensitivity and glucose tolerance via PPARγ. PPARγ is expressed in the brain, gastrointestinal tract, liver, gallbladder, kidney, reproductive tissues, and lymphoid tissues, among others (Tissue expression of PPARG - Summary - The Human Protein Atlas n.d). CBD also increases lipolysis, the metabolic process by which triglycerols break down into glycerol and free fatty acids (Caldari-Torres et al. 2023) (Table 5, Fig. 6). In the liver, PPARγ activity regulates lipid accumulation, lipid uptake, triaglycerol storage, and the formation of lipid droplets (Wang et al. 2020). In both human and mouse cultured mesenchymal stromal stem cells (MSCs), PPARγ agonism by CBD increased lipid accumulation and increased the expression of adipogenic genes, markers of adipogenic differentiation (Chang et al. 2022). Also in MSCs, CBD restores adipogenesis and chondrogenesis following lipopolysaccharide exposure (Ruhl et al. 2018). In murine skeletal muscle, PPARγ agonism increases adiponectin production and serves as a protective factor against systemic insulin resistance (Amin et al. 2010). Combined, the effects of CBD on PPARγ on metabolic outcomes may differ depending on the dose and the location of the receptor, increasing adiposity and lipid accumulation, or by impacting insulin resistance.

**Table 5:** CBD interacts with regulators of energy homeostasis

Receptor	Full receptor name	Interaction	Reference(s)
PPARγ	Peroxisome proliferator-activated receptor gamma	Agonism	O’Sullivan 2016)
GPR 3	G-protein-coupled receptor 3	Inverse agonism	Laun et al. 2019), (Laun and Song 2017)
GPR 6	G-protein-coupled receptor 6	Inverse agonism	Laun et al. 2019), (Laun and Song 2017)
TRPV1	Transient Receptor Potential Cation Channel Subfamily V Member 1	Agonism, inhibits	Muller et al. 2019; Anand et al. 2020)

CBD both activates and antagonizes TRPV1 depending on the concentration (Muller et al. 2019; Anand et al. 2020). TRPV1 regulates multiple metabolic processes depending on tissue (Luo et al. 2012). TRPV1 is expressed in the brain, liver, gallbladder, pancreas, muscle, and reproductive tissues (Tissue expression of TRPV1 - Summary - The Human Protein Atlas n.d). In the muscle, TRPV1 agonism by capsaicin increases PGC-1α expression, increases expression of genes involved in fatty acid oxidation and mitochondrial respiration, and increased oxidative fibers (Luo et al. 2012). Additionally, in vivo TRPV1 agonism enhances exercise endurance and prevents high fat diet induced metabolic disorders (Luo et al. 2012). TRPV1^−/−^ mice show decreased calcitonin gene related peptide (CGRP) production in the sensory neurons that innervate the pancreas, leading to improved insulin secretion and metabolic health (Riera et al. 2014). CBD inversely activates both GPR3 and GPR12 (Laun and Song 2017) (Table 2, Fig. 3). GPR3 and GPR12 both regulate obesity and energy balance (Bjursell et al. 2006; Godlewski et al. 2015). GPR12 knockout mice have changes in body composition, including increased body weight and fat mass, coupled with metabolic disorders including decreased respiratory exchange ratio, hepatic steatosis, and dyslipidemia (Bjursell et al. 2006). GPR3 knockout mice have late-onset obesity (Godlewski et al. 2015). These interactions suggest a potential mechanism by which CBD could improve metabolic homeostasis. Further studies are needed to understand the combinatorial effect of CBD on PPARs, TRPs, and GPCRs, as the metabolic impacts appear to be contradictory to each other. However, it is biologically plausible that differing tissues would have different responses to CBD exposure, leading to a net change or net neutral in overall metabolic efficiency.

### CBD mediates anti-inflammatory processes

**Fig. 7:**
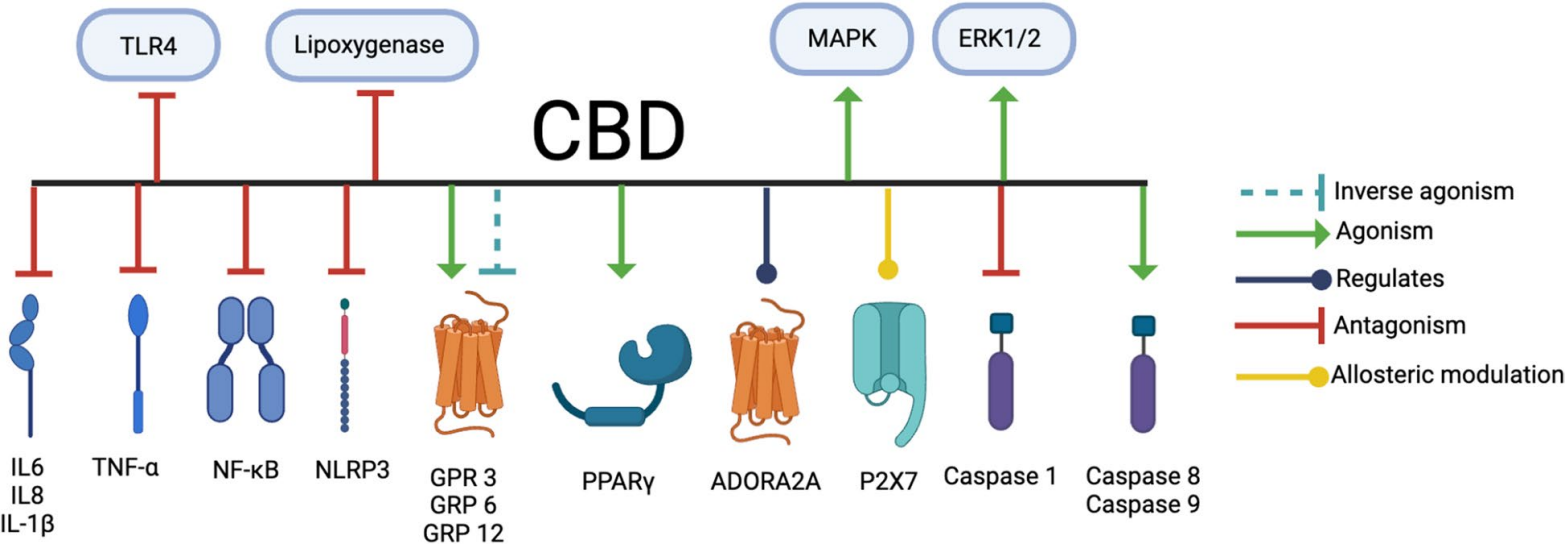
CBD impacts inflammatory and apoptotic pathways. CBD interacts with multiple components of inflammatory and apoptotic pathways. CBD antagonizes TLR4, lipoxygenase, interleukins 1α, 1β, 6, 18, TNFα, NFκB, NLRP3, and caspase 1. CBD activates caspases 8 and 9, G protein-coupled receptors GPR3, 6, and 12, PPARγ, ADORA_2A_, ERK1/2, and MAPK. CBD inversely activates GPR 3, 6, and 12, and allosterically modulates P2X7. Created with BioRender.com

CBD benefits chemotherapy patients, pain patients, and people with neurodegenerative disorders by serving as an anti-inflammatory agent (Sholler et al. 2020). As discussed by Atalay and colleagues (Atalay et al. 2019), Pereira and colleagues (Pereira et al. 2021), and Jîtcă and colleagues (Jîtcă et al. 2023), CBD inhibits reactive oxygen species (ROS) production and produces an antioxidative defense. CBD activates caspases 8 and 9 (Massi et al. 2006), which subsequently induces the intrinsic apoptotic pathways (Massi et al. 2006) (Table 6, Fig. 7). CBD antagonizes the lipoxygenase pathway (Massi et al. 2008). The lipoxygenase pathway is a pro-carcinogenic pathway which, when active, generates proinflammatory mediations including leukotrienes and lipoxins (Wisastra and Dekker 2014) (Table 6, Fig. 7).

**Table 6:** CBD impacts inflammatory and apoptotic pathways

Receptor/factor/enzyme	Full receptor/ factor/enzyme name	Interaction	Reference(s)
**Caspases**			
Caspase-1	Cysteinyl aspartate protease 1	Suppresses	Yndart Arias et al. 2023)
Caspase 8	Cysteinyl aspartate protease 8/9	Agonizes	Massi et al. 2006)
Caspase 9	Cysteinyl aspartate protease 8/9	Agonizes	Massi et al. 2006)
**Receptors**			
PPARγ	Peroxisome proliferator-activated receptor gamma	Agonizes	O’Sullivan 2016)
P2X7	P2X7	Modulates	Liu et al. 2020)
ADORA_2A_	Adenosine A_2A_ receptor	Modulates	Mecha et al. 2013)
TNF-α	Tumor necrosis factor α	Decreases	Suryavanshi et al. 2022)
NF-κB	Nuclear factor kappa B	Decreases	Chen et al. 2023)
NLRP3	Intracellular "NOD-like" receptor (NLR) proteins	Suppresses	Yndart Arias et al. 2023), (Suryavanshi et al. 2022)
TLR4	Toll like receptor 4	Decreases	Chen et al. 2023)
**G-protein-coupled receptors**			
GPR 3	G-protein-coupled receptor 3	Inversely activates, activates	Laun et al. 2019), (Laun and Song 2017)
GPR 6	G-protein-coupled receptor 6	Inversely activates, activates	Laun et al. 2019), (Laun and Song 2017)
GPR 12	G-protein-coupled receptor 12	Inverse activates, activates	Laun et al. 2019), (Laun and Song 2017)
**Interleukins**			
IL-1β*	Interleukin-1β	Decreases	Suryavanshi et al. 2022)
IL-6*	Interleukin-6	Decreases	Suryavanshi et al. 2022)
IL-8*	Interleukin-18	Decreases	Suryavanshi et al. 2022)
**MAPK Pathway**			
p38 MAPK pathway**	Mitogen activated protein kinase	Activates	Hwang et al. 2017)
ERK1/2***	Extracellular signal-regulated kinase	Activates	Vrechi et al. 2021)
Reduces both potency and efficacy of endogenous and exogenous cannabinoids on ERK1/2-PLCβ3-dependent signaling		Interaction	Laprairie et al. 2015)

^*^ IL-1 is also known as lymphocyte activating factor, endogenous pyrogen, catabolin, hemopoietin-1, melanoma growth inhibition factor, and osteoclast activating factor (Chiu et al. 2021)

^**^ P38 MAPK is also called RK or Cytokinin Specific Binding Protein (CSBP) (Yang et al. 2014)

^***^ ERK1/2 is also called MAPK42/44 (Lucas et al. 2022)

CBD antagonizes multiple pro-inflammatory processes, leading to a subsequent decrease in inflammation. CBD decreases levels of TNF-α, NF-κB, TLR4 and NLRP3 (Yndart Arias et al. 2023; Suryavanshi et al. 2022; Chen et al. 2023) (Table 6, Fig. 7), the activation of which produce proinflammatory cytokines (Blevins et al. 2022) and decreases proinflammatory cytokines IL-1β, IL-6, IL-8 (Suryavanshi et al. 2022; Dinarello 2000; Hoffmann et al. 2002; Xing et al. 1998). CBD suppresses caspase 1 (Yndart Arias et al. 2023), decreasing the pyroptosis pathway and subsequent immune cell activation (Molla et al. 2020) (Table 6, Fig. 7). CBD indirectly modulates ADORA_2A (_Mecha et al. 2013) (Table 6, Fig. 6) via inhibition of the adenosine transporter, increasing adenosine levels which can in turn activate ADORA_2A (_Pandolfo et al. 2011), which inhibits inflammation in microglia (Yuan et al. 2022). NLRP3 is suppressed by CBD (Yndart Arias et al. 2023; Suryavanshi et al. 2022) (Table 6, Fig. 7). Additionally, CBD modulates P2X7 receptors (Liu et al. 2020), which are a second signal for NLRP3 inflammasome activation and subsequent IL-1β release by decreasing calcium efflux (Table 6, Fig. 7) (Liu et al. 2020). CBD activates PPARγ (O’Sullivan 2016), which subsequently inhibits the release of inflammatory cytokines (Jiang et al. 1998) (Table 6, Fig. 7).

CBD interacts with the mitogen activated protein kinase (MAPK) pathway (Hwang et al. 2017), a signal transduction pathway that regulates gene expression, mitosis, apoptosis, and differentiation (Cargnello and Roux 2011) (Table 6, Fig. 7). CBD increases ERK1/2 and p38 activity within the MAPK pathway (Hwang et al. 2017; Vrechi et al. 2021) (Table 6, Fig. 7). ERK1/2, when activated, inhibits apoptosis and subsequently increases the rates of conversion from one cell type to another cell type (metaplasia) and increases rates of tumor development, as discussed by Mebratu and colleagues (Mebratu and Tesfaigzi 2009). Activation of p38, another component of the MAPK pathway, increases biosynthesis of proinflammatory cytokines (Xiao et al. 2002). Increased activity of both ERK1/2 and p38 increase the phosphorylation and subsequent activity of ternary complex factor (TCF) and serum response factor (SRF) (Vickers et al. 2004). Activation of TCF and SRF downregulate apoptotic pathways, similar to the effects of ERK1/2 activation (Vickers et al. 2004). In a tumor microenvironment, inhibition of apoptosis furthers tumor progression (Gadiyar et al. 2020). Additionally, CBD reduces the potency with which endogenous (2‐AG) and exogenous (THC) cannabinoids signal through the ERK1/2 pathway (Laprairie et al. 2015). CBD both activates and inversely activates GPR12 (Laun et al. 2019; Laun and Song 2017), which increases cell survival and protein kinase signaling to increase cell proliferation (Table 6, Fig. 7) (Lu et al. 2012). GPR12 agonism increases keratin 8 phosphorylation (Park et al. 2016). Phosphorylation of keratin 8 increases tumor cell migration, which contributes to metastatic capabilities of tumor cells (Busch et al. 2012). By agonizing components of the MAPK pathway and GPR12, CBD may increase tumor cell survival and migration. Henshaw and colleagues demonstrated that in vivo animal model and clinical studies validate the in vitro studies, as CBD consumption decreased proinflammatory cytokines in > 90% of studies reviewed (Henshaw et al. 2021).

### How can CBD be interacting with so many processes?

Though not yet fully mechanistically understood, there are multiple potential reasons as to why CBD is able to bind with multiple different receptors, enzymes, and ion channels in different pathways. The first primary distinction is that CBD serves as a ligand to some receptors directly, but participates in allosteric binding with many other receptors, as discussed. Previous studies demonstrate that individual ligands act as allosteric modulators for multiple receptors, dramatically increasing the number of biological effects a single ligand can have (Wang et al. 2009). Additionally, some receptors may have multiple binding sites to allow the receptor to interact with multiple ligands (Ma et al. 2002; Alhosaini et al. 2021). In this case, a smaller number of the receptor binding sites would need to be functionally able to bind to CBD in order to produce the same effect on the receptor. In consort with having multiple binding sites, some receptors are considered promiscuous receptors and regularly bind multiple ligands of different structures (Alhosaini et al. 2021; Gilberg et al. 2019). Some receptor pathways have multiple receptor subtypes or isoforms that produce the same downstream effect (Baker and Hill 2007), further increasing the likelihood that CBD could chemically interact with the pathway. When interacting with complex signal transduction pathways, CBD may indirectly induce multiple downstream effects by agonizing or antagonizing an upstream receptor. In this case, CBD may indirectly impact multiple processes without needing to directly interact with the downstream intermediates. Similarly, CBD may interact with systems that have high levels of redundancy, or similar downstream processes (Mantovani 2018). By having multiple upstream pathways induce a downstream effect, this increases the likelihood that CBD may structurally interact with one of the receptors. Lastly, some receptors may undergo conformational changes upon ligand binding (Frimurer et al. 2003). By changing the structure of the binding site, receptors may conform to a structure that CBD is capable of interacting with, only after binding of another ligand (Kondra et al. 2022). However, the morphological structure of these receptors are still being classified (Reggio 2010). As CBD gains significant traction in research, further studies are needed to understand how CBD specifically is able to interact with so many pathways.

In addition to CBD’s ability to bind with many receptors and interact with so many pathways, CBD may have an additional indirect impact on receptor activity by impacting the membrane fluidity of the cell the receptors are present on (Watkins 2019). Because CBD is highly lipophilic, it’s interactions with the lipid bilayer of cells has been under recent investigation. Nelson and colleagues propose that this impact on membrane fluidity has a direct impact on CBD’s promiscuity to receptors (Nelson et al. 2020). Watkins proposes that CBD can increase membrane fluidity, and subsequently change the conformation and gating kinetics of channels embedded in the membrane (Watkins 2019). Further studies are needed to elucidate the connections between CBD, membrane fluidity, and channel activity.

### Effects of acute CBD exposure may differ from chronic exposure

CBD may be consumed in acute settings (for example, for a sleep aid, a nausea suppressant, etc.), or chronically (Epidiolex prescriptions for seizure, etc.). Little is known about the differential effects of acute versus chronic CBD exposure. Receptors may have differential activity depending on acute or chronic exposure (Jacobson et al. 1996). For example, a receptor may activate readily upon acute exposure, though under chronic exposure, the receptor may become overactivated and subsequently become downregulated either through decreased expression levels or cell surface presentation (Posner and Laporte 2010). Conversely, other receptors may continue to signal at maximum capacity despite chronic activation (Jacobson et al. 1996). For example, when CBD activates one receptor, I may see an upregulation of downstream signaling cascades. If that receptor is downregulated, CBD may activate the receptor, but the long-term output would mimic that CBD antagonized the receptor because the receptor was downregulated and no longer signaling or weakly signaling.

### Biological effects of CBD are likely dose dependent

The activation of some CBD receptors varies depending on the dose of CBD and the affinity of CBD for the receptor (Lucas et al. 2018). For example, CBD activates TRPV1 at high concentrations (10–30 mM) and inhibits TRPV1 at low concentrations (1 mM) in varying cell culture models (Muller et al. 2019; Anand et al. 2020). For many receptors, the threshold of interaction with CBD has yet to be defined. It is possible that at low doses, CBD binds and interacts with a subset of receptors, while at high doses it interacts with a different subset of receptors in addition to high-affinity receptors. Additionally, high-dose exposure has the potential to downregulate certain receptors, leading to decreased receptor expression and activity. Because the body of research on CBD varies in methodologies (cell culture, animal model, and concentration) the effects of CBD cannot be directly compared. Because of this, not all effects mentioned are likely to be found at all doses. Further research is necessary to investigate the differential effects of CBD at standard dosing protocols to be translationally relevant.

### Consumption method affects pharmacodynamics of CBD

As discussed by Lucas and colleagues, the pharmacokinetics of CBD vary based on method of consumption (Lucas et al. 2018). Common methods of consumption of CBD include oral consumption in the form of gummies, foods, or oils, inhalation methods such as smoking or vaping, sublingual consumption of oils, topically in a lotion, or via transdermal application (Corroon and Phillips 2018). Sublingual consumption and inhalation methods have the most concentrated effect, as uptake of CBD is unimpeded (Lucas et al. 2018; Huestis 2007). Vaping products tend to be more concentrated than smoking products, leading to higher blood stream CBD levels (Lucas et al. 2018). Oral consumption of CBD products requires the CBD to undergo first pass metabolism in the liver, which causes a tenfold reduction in available CBD to be metabolized before entering the circulatory system (Franco et al. 2020). Because of this, peak metabolite concentration following oral consumption is significantly slower than that of smoking, vaping, or sublingual consumption (Lucas et al. 2018). Topical and transdermal applications lead to the lowest levels of circulating CBD and CBD metabolites (Lucas et al. 2018). In addition to the varying impact of method of consumption on pharmacokinetics, differences in metabolism and binding of CBD may differ from CBD metabolites. However, in the context of receptor activation, few studies elucidate the differential impact of CBD from the major metabolites, including 7-OH CBD, CBD-glucuronide, and 10-OH-7-COOH-CBD (Ujváry and Hanuš 2016). As each metabolite varies slightly in structure, receptor binding ability or affinities may differ (Ujváry and Hanuš 2016). These many metabolites may contribute to the mechanism by which CBD acts on such a wide variety of receptors, as each metabolite has a slightly different structure and can therefore interact as ligands to receptors with different binding sites.

### Challenges and Limitations

CBD research faces several significant challenges that complicate the interpretation and application of findings. One major limitation is the difficulty in sourcing high-quality and standardized CBD for research purposes, which hinders replication and consistency across studies. Additionally, basic science and preclinical studies vary widely in dosing regimens and routes of administration, making it difficult to compare findings or translate them to human applications. In human studies, variability in cannabinoid formulations—ranging from pure CBD isolates to full-spectrum extracts with other cannabinoids—further complicates comparisons across trials.

A critical translational gap exists between in vitro and in vivo research, as many reported effects may not be achievable at physiologically relevant doses in humans. The lack of standardized dose–response studies makes it difficult to determine whether findings from basic science research hold clinical significance. Additionally, research often fails to distinguish between acute and chronic exposure, limiting our ability to predict long-term outcomes. Existing studies have tested a broad range of doses, from low doses (~ 5–25 mg/day) used in wellness products to high doses (300–1,500 mg/day) investigated in clinical trials for conditions such as epilepsy and anxiety. However, data on the effects of chronic, moderate-dose CBD use remain limited. Addressing these limitations requires carefully designed studies that evaluate CBD’s pharmacokinetics, bioavailability, and sustained effects across different dosing regimens and patient populations.

### Future directions and implications for clinical practice

Future research must bridge the gap between preclinical findings and human applications by ensuring translational relevance in dosing, administration routes, and outcome measures. Studies should clearly document the sourcing and composition of CBD formulations to improve reproducibility and clinical applicability. Additionally, making research findings widely accessible is essential, as clinicians and researchers across various disciplines need accurate and transparent data to guide patient care. This is particularly important given that patients may use CBD off-label, recreationally, or as a prescribed treatment, necessitating a comprehensive and evidence-based understanding of its effects across different populations. For example, research must define safe co-administration guidelines and identify potential risks associated with long-term CBD use in polypharmacy settings given the impact on CYP enzymes. Given the widespread use of CBD across different patient populations, future studies should prioritize personalized CBD therapy, evaluating how genetic, metabolic, and environmental factors influence individual responses. Long-term safety trials are essential to guide clinical recommendations, regulatory policies, and patient education. Standardizing research methodologies, ensuring transparent reporting, and making findings accessible to healthcare providers will be key to integrating CBD into evidence-based clinical practice.

## Conclusion

CBD is rapidly gaining traction both in the pharmaceutical industry and as a widely available supplement to aid common ailments like nausea or insomnia, to rare conditions like childhood epilepsy (Abu-Sawwa et al. 2020; Data and Statistics. April 22 2022). As CBD consumption is not regulated, patients may co-consume CBD with pharmaceutical medications. CBD’s interaction with multiple body systems, and its effects on drug metabolism pose potential risks to unsuspecting patients. Clinicians and clinical researchers should ask patients about CBD consumption and should educate patients on potential drug-drug interactions. This review compiles the many processes that CBD interacts with that can confer multiple impacts, including affecting nausea, insomnia, seizure, sex hormone regulation, drug metabolism, and inflammation. While CBD has many beneficial effects, many of these interactions also have the potential to confer harm, meaning that CBD consumption should be monitored, especially when co-consumed with pharmaceutical or recreational substances. Further research is needed to understand the interactions between the processes included herein, and the translation from cell culture or animal model studies into human consumption through clinical research studies.

## Data Availability

No datasets were generated or analysed during the current study.

## Abbreviations

2-AG: 2-Arachidonoylglyerol
5HT_1A_: 5-Hydroxytryptamine receptor 1A
5HT_3A_: 5-Hydroxytryptamine receptor 3A
17OHP: Progesterone 17-hydroxylase
ACOG: American College of Obstetrics and Gynecology
ADORA_2A_: Adenosine A_2A_ receptor
AEA: Anandamide
Caspase: 1-Cysteinyl aspartate protease 1
cAMP: Cyclic adenosine monophosphate
CBD: Cannabidiol
CBDV: Cannabidivarin
CBC: Cannabichromene
CBCV: Cannabichromevarin
CBG: Cannabigerol
CBDA: Cannabidioloic acid
CBGA: Cannabigerolic acid
CBGV: Cannabigerovarin
CBN: Cannabinol
CBNA: Cannabinolic acid
CB_1_: Cannabinoid receptor type 1
CB_2_: Cannabinoid receptor type 2
CPS: Child Protective Services
CYP: Cytochrome p450 enzyme
CYP3A5: Cytochrome P450 3A5
CYP3A7: Cytochrome P450 3A7
CYP3A4: Cytochrome P450 3A4
CYP2C9: Cytochrome P450 2C9
CYP1A1: Cytochrome P450 1A1
CYP1A2: Cytochrome P450 1A2
CYP1B1: Cytochrome P450 1B1
CYP2D6: Cytochrome P450 2D6
CYP2C19: Cytochrome P450 2C19
CYP2B6: Cytochrome P450 2B6
CYP2J2: Cytochrome P450 2J2
D2: Dopamine receptors
ERK1/2: Extracellular signal-regulated kinase
FAAH: Fatty acid amine hydrolase
FDA: Food and Drug Administration
GPR55: G protein-coupled receptor 55.
GlyRs: Ligand-gated glycine receptors
GPR3: G protein-coupled receptor 3.
GPR6: G protein-coupled receptor 6.
GPR12: G protein-coupled receptor 12
GPX: Glutathione peroxidase
IDO1/2: Indoleamine-pyrrole 2,3-dioxygenase
IL-1β: Interleukin-1β
IL-6: Interleukin-6
IL-8: Interleukin-8
KV7.2/3: Potassium voltage-gated channel subfamily KQT member 2 and 3
MAPK: Mitogen activated protein kinase
NaV_1_: Sodium channel protein type 1 subunit
NF-κB: Nuclear factor kappa B
NIDA: National Institute of Drug Abuse
NLRP3: Intracellular “NOD-like” receptor (NLR) family pyrin domain containing 3
NVP: Nausea and vomiting in pregnancy
PGC-1α: Peroxisome proliferator-activated receptor gamma coactivator 1-alpha
PPARα: Peroxisome proliferator-activated receptor alpha
PPARγ: Peroxisome proliferator-activated receptor gamma
THC: Tetrahydrocannabinol
THCA: Tetrahydrocannabinolic acid
THCV: Tetrahydrocannabivarin
TRPA1: Transient receptor potential cation channel subfamily A member 1
TRPM8: Transient receptor potential cation channel subfamily M member 8
TRPV1: Transient receptor potential cation channel subfamily V member 1
TRPV2: Transient receptor potential cation channel subfamily V member 2
TNF-α: Tumor necrosis factor α
TLR4: Toll like receptor 4
